# Measurement of green total factor productivity on Chinese laying hens: From the perspective of regional differences

**DOI:** 10.1371/journal.pone.0255739

**Published:** 2021-08-05

**Authors:** Junzhi Li, Junwei Li, Zhenlei Sun, Shen Zhong

**Affiliations:** 1 School of Public Administration, Jilin University, Changchun, Jilin, PR China; 2 School of Management, Inner Mongolia University for Nationalities, Tongliao, Inner Mongolia, PR China; 3 School of Finance, Harbin University of Commerce, Harbin, Heilongjiang, PR China; 4 College of Physical Education, Inner Mongolia University for Nationalities, Tongliao, Inner Mongolia, PR China; Universidad Nacional Autonoma de Nicaragua Leon, NICARAGUA

## Abstract

Eggs contain the essential cholesterol and protein for the human body, which plays an irreplaceable role in human survival, production and life. There are significant differences in the development of laying hens feeding in different regions. It is of great significance to improve egg production and reduce pollution emission for China’s laying hens industry. Based on the SBM model, this paper constructs MML index, considering unexpected output under common frontier, to comprehensively evaluate the green total factor productivity on Chinese laying hens (GTCL). The results show that: (1) GTCL shows a large spatial and temporal differentiation under both the common frontier and the regional frontier. Compared with the eastern region and central region, the western region has obvious advantages in GTCL. (2) GTCL overall shows a downward trend, however, it emerges an upward trend in recent years. (3) Compared with small-scale and large-scale, middle-scale GTCL has advantages. According to the above empirical results, combined with the China’s actual national situation, this paper finally puts forward some policy recommendations to improve GTCL.

## Introduction

Eggs are an important source of protein intake for urban and rural residents [1,2]. The laying hens breeding industry is the pillar industry of animal husbandry in China [3,4]. According to the data of National Bureau of Statistics of China (NBSC), China’s egg production increased steadily from 21.347 million tons in 1999 to 31.2828 million tons in 2018. Although the production is large, it is still unable to meet the huge demand of Chinese people for eggs. In order to narrow the gap between supply and demand, improving the efficiency of domestic laying hens breeding is necessary. It can not be ignored that chemical oxygen demand (COD), total nitrogen (TN) and total phosphorus (TP) will be produced in the process of layer breeding. In order to comply with the global sustainable development plan and goal, this paper will measure the green total factor productivity on Chinese laying hens (GTCL).

There are two main phenomena in the process of laying hens breeding in China: Firstly, there are obvious regional differences in laying hens breeding in different regions [5,6]. The geographical climate, water and soil conditions, and the distance to the corn belt are diverse in different regions and provinces [7,8]. In addition, feed prices, transportation conditions and local government support for laying hens breeding vary from region to region [9–12]. The eastern region is densely populated with small land area, lacking natural conditions for laying hens breeding; the central region is located in the middle of China, with superior geographical position and low transportation cost; the western region has low labor cost, large area, open terrain and superior natural conditions. Second, pollutants will be generated during laying hens breeding [13–15]. In 2015, the No.1 Central Document clearly states that “Strengthen the treatment of agricultural non-point source pollution, carry out regional demonstration of resource utilization of livestock and poultry manure, and vigorously promote the development of agricultural circular economy.” According to the Ministry of Agriculture “Regulations on Pollution Control of Livestock and Poultry Scale Farming”, the current annual output of livestock and poultry manure in China is about 3.8 billion tons, but the comprehensive utilization rate is less than 60%. Livestock and poultry wastewater are not only large in quantity, but also high in pollutant concentration [16–18]. Improper treatment will cause serious deterioration of surface or underground water quality [19–21]. Thus it can be seen that it is necessary to consider regional differences and environmental factors in the process of laying hens breeding. While the existing literature makes great contributions, it does not take into account the regional heterogeneity and the influence of unexpected output. Therefore, this paper uses SBM-MML (Slack Based Measure—Metafrontier Malmquist Luenberger) model and adds negative output under the condition of considering the regional heterogeneity to conduct a comprehensive empirical analysis on GTCL from 2004 to 2018.

The rest part of this paper is arranged as follows: the second part introduces the research situation of the relevant literature; the third is the relevant theoretical basis, and explains the variable selection and data sources; Empirical analysis is in the fourth part; and the fifth is conclusions and relevant policy recommendations.

## Literature review

Among the existing researches on agricultural efficiency, there are few on the production efficiency of laying hens. Huang and Bagi (1984) and Kalirajan (1991) studied the rice production efficiency in India, and found that there was no correlation between the production scale and the loss of technical efficiency [22,23]. Ekanayske and Jayasuriya (1987) measured rice production efficiency in Sri Lanka and found that labor quality and capital deepening had a significant impact on it [24]. Chavas and Aliber(1993) conducted a nonparametric analysis on the cost efficiency of livestock and poultry raising in 545 farms in Wisconsin in 1987. They found that the efficiency of the samples ranged from 0.76 to 0.96, and there was a certain efficiency loss, which was mainly caused by the inefficient allocation of factors and the diseconomy of scale [25]. Fang and Fabiosa (2002) pointed out that there are three modes of pig breeding in China, that is, small-scale scattered breeding, medium-scale breeding and large-scale breeding, the breeding cycle of scattered breeding was the longest, and the cost of specialized breeding was lower than the cost of large-scale breeding [26]. Perez et al. (2007) studied the production efficiency of Spanish mutton sheep, and they found that the key to improve the production efficiency of Spanish mutton sheep is to strengthen management [27]. Fogarasi (2008) pointed out that the transformation of mutton sheep farming from extensive management to intensive management in the world benefited from the improvement of mutton sheep production efficiency. With the expansion of breeding scale, the breeding efficiency is also improved [28]. Zúniga-González (2011) analyzed the technical efficiency of small farms’ organic fertilizer in Nicaragua from 1998 to 2005, and found that the scale efficiency of organic fertilizer decreased during the sample period, and the average technical efficiency was less than 0.62 [29]. Reddy (2012) compared the production of crops between Orissa and India’s other regions, and found that the low growth rate of agriculture in Orissa was due to its rice-dominated agricultural products and low diversification of rice cultivation [30]. Even if there are researches on laying hens, there are few researches on production efficiency. Sugiyama (1987) analyzed Taiwan’s livestock and poultry industry, especially laying hens industry, based on the statistics of Taiwan government from 1975 to 1984, and compared it with Japanese breeding industry. He pointed out that it was necessary to improve the situation of poultry seedling trading market, pay attention to the cultivation of poultry talents, and change the breeding mode [31]. Samarendu and Rajendran (2003) studied the data of 2000, and pointed out that one of the important factors hindering the development of laying hens breeding industry was the lack of an effective marketing system. The collection, storage, processing and sales system of eggs and poultry meat is not perfect, especially in rural areas, which seriously restricted the development of domestic poultry breeding industry [32]. Few studies on the production efficiency of laying hens did not take into account the regional heterogeneity. Ojo (2003) calculated the technical efficiency of laying hens production in Nigeria by collecting the data of 200 farms, and found that the efficiency value was between 0.239–0.933. And the larger the scale of breeding, the higher the production efficiency. But he did not consider the impact of environmental factors on production efficiency [33]. Yusuf and Malomo (2007) calculated the technical efficiency of laying hens production in Ogun State, and obtained that the efficiency of sample farmers was 0.888. Similarly, the larger the scale, the higher efficiency it is [34]. Environmental factors are not taken into account as well.

With the rapid development of agricultural economy, the resource and environmental problems restrict the economic development, which makes people realize that people cannot blindly pursue speed in the process of development, and sustainable development is the only way for agricultural economic development. Therefore, more scholars have studied the total factor productivity (TFP) of agriculture under the constraints of resources and environment, and they point out that resources and environment are not only endogenous variables of agricultural economic development, but also rigid constraints of its development. Obviously, in order to accurately evaluate agricultural economic performance and development of agricultural economy through TFP, it needs to coordinate resources and environment factors with traditional capital labor factors. Kasimati (2003) proposed that energy factor, capital factor and labor factor should be introduced in the production function, and green total factor productivity (GTFP) should be calculated on the basis of TFP [35]. Ramanathan (2005) proposed that in the study of agricultural TFP, pollution emission is calculated as an unpaid input. In the later research, the scholar found that the pollution emission has the characteristics of output. Therefore it is not feasible to put pollution emission into input, which should be included in the unexpected output [36]. Caves et al. (1982) used mathematical method to deduce the Tornqvist index into Malmquist index, and used Malmquist index to measure TFP for the first time [37]. Fare et al. (1994) put forward a multi-period productivity analysis method, on the basis of Caves’ research, which can change with time, that is, DEA-Malmquist (Data Envelopment Analysis- Malmquist) index method which can be used to measure panel data [38]. Zúniga-González (2020) uses DEA-Malmquist index to measure agricultural TFP of 14 developing countries, the results show that TFPs of Cuba and other five places are less than the average growth rate of the sample (3.9%) [39]. Chung et al. (1997) proposed for the first time that pollution emission should be regarded as the unexpected output to measure the TFP of pulp mills in Sweden [40]. Through the empirical analysis of the directional distance function (DDF) and Malmquist index, it was found that the fitting of the impact of pollution emission factors on economic growth makes the TFP more comprehensive and reasonable. The idea of reflecting environmental pollution factors as unexpected output into the calculation of TFP has gradually become a mainstream idea in the study of TFP, which is adopted by Dwyer et al. (2005), Hailu and Veeman (2015), Ananda and Hampf (2015), Li and Lin (2015), Dios-Palomares et al. (2015) [41–45]. Considering resource and environment constraints, agricultural TFP takes environmental pollution as unexpected output and resource consumption as input for accounting. Therefore, this idea is adopted in the study of GTCL.

Hjalmarsson et al. (1996) used the traditional DEA and Stochastic Frontier Analysis (SFA) methods to estimate the production efficiency of Columbia cement plant, and found that the efficiency values calculated by the model showed the same change trend [46]. Koetter et al. (2006) used the traditional DEA and SFA methods to calculate the cost efficiency of 34192 German banks from 1993 to 2004. It was found that the cost efficiency calculated by SFA method was higher than that calculated by DEA method, and DEA method was more sensitive to outliers [47]. SFA will produce random measurement error, and its calculation results are easily affected by the selection of influencing factors [48]. The traditional radial DEA model only considers the proportion improvement of all inputs or outputs when evaluating the efficiency of the decision-making unit (DMU). That is, the invalid DMU achieves the frontier by reducing all inputs or increasing all outputs in the same proportion, while ignoring the possible non-proportion improvement, namely relaxation improvement [49]. Although Charnes et al. (1978) put forward the additive DEA model to consider input and output slack in efficiency measurement, the model can only divide all DMUs into effective and ineffective types according to the size of slack, and cannot further obtain the specific efficiency value of each DMU [50]. Therefore, Tone (2002) proposed the Slack Based Measure (SBM) model, which takes the input and output slack into account when evaluating the efficiency of DMU [51]. The SBM model opens another new direction of DEA model research. This paper uses the SBM model to measure GTCL.

To sum up, the innovation of this paper is mainly reflected in the following aspects: (1) In the research subject, this paper studies the feeding efficiency of Chinese laying hens, and there are few related literature. This research subject is innovative. (2) In variable selection and index calculation, unexpected output is added, and different weights are given to COD, TN and TP to calculate the total pollution emissions. (3) In terms of research methods, considering the regional heterogeneity, this paper constructs the SBM-MML model based on the common frontier to evaluate GTCL of different scales.

## Methodology

### SBM model based on the common frontier

The calculation principle of SBM (Slack Based Measure) model is as follows:

$$\begin{array}{l} \delta =\min\frac{1-\frac{1}{N}\sum_{i=1}^{N}\frac{s_{ik}^{-}}{x_{ik}}}{1+\frac{1}{M}\sum_{r=1}^{M}\frac{s_{rk}^{+}}{y_{rk}}}\\ s.t.\left\{ \begin{array}{l} \sum_{z=1}^{Q}\chi_{z}x_{iz}+s_{ik}^{-}=x_{ik},i=1,\cdots ,N;\\ \sum_{z=1}^{Q}\chi_{z}y_{rz}-s_{rk}^{+}=y_{rk},r=1,\cdots ,M;\\ \chi_{z}\geq 0,z=1,\cdots ,Q;\\ s_{ik}^{-}\geq 0,s_{rk}^{+}\geq 0,\forall i,r \end{array}\right. \end{array}$$ (1)

Where, *δ* is the efficiency of DMU, and the optimal solution of the model is $\left(\delta ,s_{ik}^{-*},s_{rk}^{+*},\chi_{z}^{*},\forall i,r,k\right)$. $s_{ik}^{-*}$ and $s_{rk}^{+*}$ represents the input slack and output slack of DMU respectively. The larger the efficiency value *δ* of the objective function is, the higher the efficiency value of the evaluated DMU is. When *δ* = 1, that is $s_{ik}^{-*}=s_{rk}^{+*}=0,\forall i,r$, the evaluated DMU is called SBM effective. *N* and *M* represent the number of input variables and output variables, respectively. *χ*_*z*_ is the weight of DMU Z when constructing the environment technology structure. *x* and *y* are input and expected output vectors, respectively.

Cooper et al. (2007) combined SBM model with environmental technology to establish SBM model considering environmental factors [52]. It can be written as:

$$\begin{array}{l} \delta =\min\frac{1-\frac{1}{N}\sum_{i=1}^{N}\frac{s_{ik}^{-}}{x_{ik}}}{1+\frac{1}{M+H}(\sum_{r=1}^{M}\frac{s_{rk}^{+}}{y_{rk}}+\sum_{a=1}^{H}\frac{s_{ak}^{-}}{f_{ak}})}\\ s.t.\left\{ \begin{array}{l} \sum_{z=1}^{Q}\chi_{z}x_{iz}+s_{ik}^{-}=x_{ik},i=1,\cdots ,N,s_{ik}^{-}\geq 0;\\ \sum_{z=1}^{Q}\chi_{z}y_{rz}-s_{rk}^{+}=y_{rk},r=1,\cdots ,M,s_{rk}^{+}\geq 0;\\ \sum_{z=1}^{Q}\chi_{z}f_{az}+s_{ak}^{-}=f_{ak},a=1,\cdots ,H,s_{ak}^{-}\geq 0;\\ \chi_{z}\geq 0,z=1,\cdots ,Q;\forall i,r,a \end{array}\right. \end{array}$$ (2)

Where *N*, *M* and *H* represent the number of inputs, expected output and unexpected output variables respectively. $s_{ik}^{-*}$、 $s_{rk}^{+*}$ and $s_{ak}^{-*}$ represent the input relaxation, expected output relaxation and unexpected output relaxation of DMU, respectively. *x*、 *y* and *f* are input vector, expected output vector and unexpected output vector respectively.

Hayami (1969) first proposed the preliminary concept of common frontier, which is more suitable for examining the input-output relationship between different categories at the same time [53]. The SBM models based on common frontier and inter-group scale frontier are as follows:

$$\begin{array}{l} \delta^{M}=\min\frac{1-\frac{1}{N}\sum_{t=1}^{T}\sum_{i=1}^{N}\frac{s_{ik}^{-}}{x_{{}_{ik}}^{t}}}{1+\frac{1}{M+H}(\sum_{t=1}^{T}\sum_{r=1}^{M}\frac{s_{rk}^{+}}{y_{{}_{rk}}^{t}}+\sum_{t=1}^{T}\sum_{a=1}^{H}\frac{s_{ak}^{-}}{f_{{}_{ak}}^{t}})}\\ s.t.\left\{ \begin{array}{l} \sum_{t=1}^{T}\sum_{z=1}^{Q_{M}}\alpha_{{}_{z}}^{t}x_{{}_{iz}}^{t}+s_{ik}^{-}=x_{{}_{ik}}^{t},i=1,\cdots ,N,s_{ik}^{-}\geq 0;\\ \sum_{t=1}^{T}\sum_{z=1}^{Q_{M}}\alpha_{{}_{z}}^{t}y_{{}_{rz}}^{t}-s_{rk}^{+}=y_{{}_{rk}}^{t},r=1,\cdots ,M,s_{rk}^{+}\geq 0;\\ \sum_{t=1}^{T}\sum_{z=1}^{Q_{M}}\alpha_{{}_{z}}^{t}f_{{}_{az}}^{t}+s_{ak}^{-}=f_{{}_{ak}}^{t},a=1,\cdots ,H,s_{ak}^{-}\geq 0;\\ \alpha_{{}_{z}}^{t}\geq 0;t=1,\cdots ,T;z=1,\cdots ,Q_{M};\forall i,r,a \end{array}\right. \end{array}$$ (3)

$$\begin{array}{l} \delta^{G}=\min\frac{1-\frac{1}{N}\sum_{t=1}^{T}\sum_{i=1}^{N}\frac{s_{ik}^{-}}{x_{{}_{ik}}^{t}}}{1+\frac{1}{M+H}(\sum_{t=1}^{T}\sum_{r=1}^{M}\frac{s_{rk}^{+}}{y_{{}_{rk}}^{t}}+\sum_{t=1}^{T}\sum_{a=1}^{H}\frac{s_{ak}^{-}}{f_{{}_{ak}}^{t}})}\\ s.t.\left\{ \begin{array}{l} \sum_{t=1}^{T}\sum_{z=1}^{Q_{G}}\beta_{{}_{z}}^{t}x_{{}_{iz}}^{t}+s_{ik}^{-}=x_{{}_{ik}}^{t},i=1,\cdots ,N,s_{ik}^{-}\geq 0;\\ \sum_{t=1}^{T}\sum_{z=1}^{Q_{G}}\beta_{{}_{z}}^{t}y_{{}_{rz}}^{t}-s_{rk}^{+}=y_{{}_{rk}}^{t},r=1,\cdots ,M,s_{rk}^{+}\geq 0;\\ \sum_{t=1}^{T}\sum_{z=1}^{Q_{G}}\beta_{{}_{z}}^{t}f_{{}_{az}}^{t}+s_{ak}^{-}=f_{{}_{ak}}^{t},a=1,\cdots ,H,s_{ak}^{-}\geq 0;\\ \beta_{{}_{z}}^{t}\geq 0;t=1,\cdots ,T;z=1,\cdots ,Q_{G};\forall i,r,a \end{array}\right. \end{array}$$ (4)

*T* is the number of years. *Q*_*M*_ and *Q*_*G*_ represent the number of DMU under the common frontier and inter-group frontier, respectively. *α* and *β* are the intensity variables under the common frontier and inter-group frontier, respectively.

### Metafrontier-Malmquist-Luenberger index and its decomposition

DEA is a nonparametric method, which cannot calculate TFP of two periods, but its TFP index can be calculated, that is $MI_{t-1}^{t}$. All input and output data during the sample period are taken as the reference set of the current period. Using the global DEA method to construct the production frontier, the GTCL and its decomposition indexes are calculated under the conditions of common frontier and group frontier respectively. On the basis of Chung et al. (1997) [40], Oh (2010) constructed the Metafrontier-Malmquist-Luenberger (MML) index [54]. The MML index takes the sum of all phases as the reference, and each phase refers to the same frontier. All the evaluated DMUs are included in the global reference set, which is expressed as follows:

$$M^{G}(x)=M^{1}(x^{1})\cup M^{2}(x^{2})\cup \ldots \cup M^{T}(x^{T})$$ (5)

$$M^{t}(x^{t})=\{(y^{t},f^{t})|x^{t}\;can\;produce\;(y^{t},f^{t})\}$$ (6)

The changes of GTCL were analyzed from a global perspective. This paper selects the MML index as the GTCL. At the same time, in order to further explore the sources of GTCL changes, this paper further decomposes MML index into efficiency change (EC) index and technology change (TC) index. The value of EC mainly refers to the improvement of resource allocation efficiency and management system, while the value of TC mainly refers to the improvement of production technology. According to Wang et al. (2013) [55], the formula of MML index is as follows:

$$\begin{array}{l} MML_{t-1}^{t}=\sqrt{\frac{1-M_{t-1}(x^{t},y^{t},f^{t};y^{t},-f^{t})}{1-M_{t-1}(x^{t-1},y^{t-1},f^{t-1};y^{t-1},-f^{t-1})}\times \frac{1-M_{t}(x^{t},y^{t},f^{t};y^{t},-f^{t})}{1-M_{t}(x^{t-1},y^{t-1},f^{t-1};y^{t-1},-f^{t-1})}}\\ =\sqrt{\frac{1-M_{t-1}(x^{t-1},y^{t-1},f^{t-1};y^{t-1},-f^{t-1})}{1-M_{t}(x^{t-1},y^{t-1},f^{t-1};y^{t-1},-f^{t-1})}\times \frac{1-M_{t-1}(x^{t},y^{t},f^{t};y^{t},-f^{t})}{1-M_{t}(x^{t},y^{t},f^{t};y^{t},-f^{t})}}\\ \times \frac{1-M_{t}(x^{t},y^{t},f^{t};y^{t},-f^{t})}{1-M_{t-1}(x^{t-1},y^{t-1},f^{t-1};y^{t-1},-f^{t-1})}=TC_{t-1}^{t}\times EC_{t-1}^{t} \end{array}$$ (7)

The input, desired output and undesired output of the period from t-1 to t are expressed as (*x*^*t*−1^,*y*^*t*−1^,*f*^*t*−1^) and (*x*^*t*^,*y*^*t*^,*f*^*t*^). $TC_{t-1}^{t}$ represents the contribution of DMU’s technological progress from t-1 to t for the improvement of GTCL. $EC_{t-1}^{t}$ represents the contribution of DMU’s technical efficiency improvement from t-1 to t for GTCL. The larger the value is, the greater the contribution is. The result of MML index is MI. Under the common frontier and group frontier, GTCLs are as follows:

$$metaMI_{t-1}^{t}=\sqrt{\frac{1-M_{t-1}^{m}(x^{t},y^{t},f^{t};y^{t},-f^{t})}{1-M_{t-1}^{m}(x^{t-1},y^{t-1},f^{t-1};y^{t-1},-f^{t-1})}\times \frac{1-M_{t}^{m}(x^{t},y^{t},f^{t};y^{t},-f^{t})}{1-M_{t}^{m}(x^{t-1},y^{t-1},f^{t-1};y^{t-1},-f^{t-1})}}$$ (8)

$$groupMI_{t-1}^{t}=\sqrt{\frac{1-M_{t-1}^{g}(x^{t},y^{t},f^{t};y^{t},-f^{t})}{1-M_{t-1}^{g}(x^{t-1},y^{t-1},f^{t-1};y^{t-1},-f^{t-1})}\times \frac{1-M_{t}^{g}(x^{t},y^{t},f^{t};y^{t},-f^{t})}{1-M_{t}^{g}(x^{t-1},y^{t-1},f^{t-1};y^{t-1},-f^{t-1})}}$$ (9)

For the DMUs with regional heterogeneity, the regional gap between group frontier and common frontier can be calculated, which is caused by specific group institutional structure. The basic idea of this method is: Under the same input factors, the common boundary and group boundary are constructed, the environmental efficiency values under the common frontier and group frontier are calculated respectively, and the TGR (technology gap ratio) of the DMUs under the common frontier and group frontier scale is obtained. The formula is as follows:

$$TGR=\frac{\delta^{M}}{\delta^{G}}$$ (10)

*δ*^*M*^ is the environmental efficiency value under the common frontier. *δ*^*G*^ is the environmental efficiency value under the group frontier. TGR is used to measure the distance between the optimal production technology and the potential optimal technology of the group, and whether there are differences in GTCL under different groups. The closer the TGR is to 1, the closer the technology level of this region is to the best potential technology level. Conversely, the farther the TGR is away from 1, the greater the gap between the technology level and the potential best technology level of this region is.

### Description and sources of data

Based on the existing literature, this paper selects five indicators to build the input-output index system. The details are as follows:

1. Number of concentrates. It mainly includes the crop seeds and the by-products of their processing.
2. Number of food consumption. The amount of food consumed is the amount of food consumed by laying hens. For example: wheat, barley, wheat bran, corn, sorghum, broken rice, etc.
3. Material costs. It is obtained by the sum of labor expenses, water and fuel power expenses and medical and epidemic prevention expenses. Labor expenses refers to the human management expenses required for each egg chicken from the embryonic stage to the mature stage to the laying stage. Water and fuel power expenses include water, electricity, coal and other fuel power expenses. Medical epidemic prevention expenses include the expenses of disease prevention and treatment.
4. Main product production. It is a positive output, which is the egg production per laying hens.
5. Total discharge. It is an unexpected output. According to the method of *The Manual of Pollutant Discharge Coefficient*, Eq (11) is applied to calculate the COD, TN and TP of each laying hen. In addition, according to the method of class GB3838-2002 water quality standard in V, Eq (12) is applied to calculate the total discharge.

Pollutionemissions=Coe×Days (11)

$$\mathrm{Total}\;\mathrm{discharge}=\frac{\mathrm{COD}}{40}+\frac{\mathrm{TN}}{2}+\frac{\mathrm{TP}}{0.4}$$ (12)

Where Coe is the pollution discharge coefficient and the Days is the average breeding days.

The data in this paper is from 2004–2018 *“National Agricultural Product Cost and Benefit Data Compilation”* and the first national pollution source census leading group office issued *“Pollution Discharge Coefficient Manual”*. The number of concentrates, the number of foods consumed, labor expenses, water and fuel power expenses, medical and epidemic prevention expenses, main product production, and average breeding days all come from *“National Agricultural Product Cost and Benefit Data Compilation”*. The pollution discharge coefficient of laying hens is derived from “*The Manual of Pollutant Discharge Coefficient”*.

Based on the data of 24 major egg producing provinces (municipalities) in China from 2004 to 2018, according to the above two data on the definition of scale, the types of laying hens breeding are divided into three scales: 300–1000 for small scale, 1000–10000 for middle scale, and more than 10000 for large scale. Eliminate the singular data in the three scales and use the average method to make up for the missing data. The remaining small-scale groups are Liaoning, Shandong, Henan, Heilongjiang, Jilin, Shanxi and Shaanxi. The remaining middle-scale groups are Beijing, Hebei, Jiangsu, Liaoning, Shandong, Tianjin, Zhejiang, Anhui, Henan, Heilongjiang, Jilin, Hubei, Inner Mongolia, Shanxi, Yunnan, Gansu, Ningxia, Shaanxi, Sichuan, Xinjiang and Chongqing. The remaining large-scale groups are Beijing, Fujian, Guangdong, Henan, Jiangsu, Liaoning, Shandong, Tianjin, Anhui, Hainan, Heilongjiang, Hubei, Jilin, Shanxi, Yunnan, Gansu, Sichuan and Chongqing. The above provinces are divided into three regions: Eastern Region (Liaoning, Shandong, Beijing, Hebei, Jiangsu, Tianjin, Zhejiang, Fujian, Guangdong, Hainan); Central Region (Henan, Heilongjiang, Jilin, Shanxi, Anhui, Hubei, Inner Mongolia); and Western Region (Shaanxi, Gansu, Ningxia, Sichuan, Xinjiang, Chongqing, Yunnan). The final division of sample provinces is shown in Table 1.

**Table 1:** Samples division from 2004–2018.

	Small scale	Middle scale	Large scale
Eastern Area	Shandong, Liaoning	Jiangsu, Liaoning, Beijing, Tianjin, Hebei, Zhejiang, Shandong	Jiangsu, Liaoning, Beijing, Tianjin, Shandong, Hainan, Guangdong, Fujian
Central Area	Heilongjiang, Jilin, Henan, Shanxi	Heilongjiang, Jilin, Henan, Shanxi, Hubei, Anhui, Inner Mongolia	Heilongjiang, Jilin, Henan, Shanxi, Hubei, Anhui
Western Area	Shaanxi	Shaanxi, Ningxia, Gansu, Yunnan, Sichuan, Chongqing, Xinjiang	Gansu, Yunnan, Sichuan, Chongqing

## Results and discussions

### The overall change of GTCL in China

**Fig 1:**
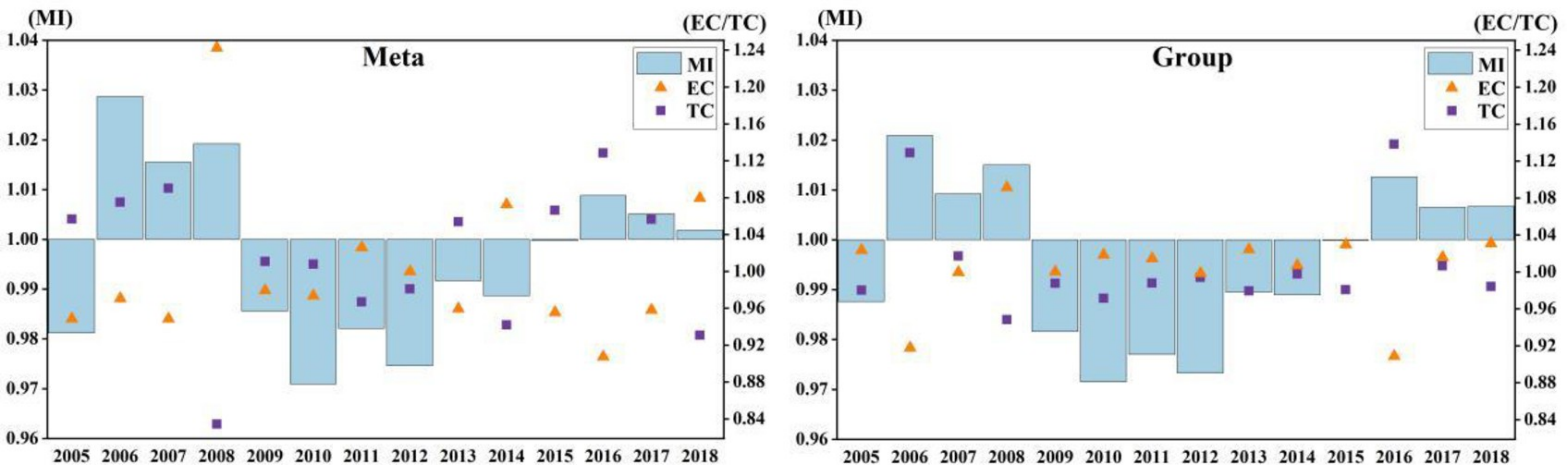
China’s GTCL and its decomposition indexes during 2004–2018.

It can be seen from Fig 1 that the fluctuation trends under the common frontier and the group frontier are basically the same, but the fluctuation ranges are different. GTCL shows negative growth in most years, in 2006 GTCL reached the highest value, with an increase of 2.87% under the common frontier and 2.09% under the group frontier. This is because since 2006, the profit of laying hens breeding has gradually increased, and the breeding enthusiasm of farmers has gradually improved. The global financial crisis broke out in 2008, which turned GTCL from positive growth to negative growth. It indicated that the influence of external environment and the stability of the market had an essential impact on laying hens’ production. In 2010, GTCL was the lowest, with a decline of 2.93% under the common frontier and 2.85% under the group frontier. This is because in 2010, the central government began to support the standardized scale breeding farms of laying hens by the way of “awards instead of subsidies”, and started the demonstration and creation activities of livestock and poultry standardization. However, at present, the laying hens breeding is still dominated by professional family farming and small-scale breeding in China [56,57]. Therefore, the GTCL in 2010 was the lowest, indicating that the government’s policies will have an important impact on China’s laying hens breeding industry.

Compared with the pig industry, the development of China’s laying hens industry is relatively slow. This is mainly because of the lack of scale factories, especially the problem of the industry itself. First of all, the scale economy is not economical. Theoretically, the more laying hens are raised, the greater the scale effect and the lower the cost. However, that is not the case in China, the actual situation is that the cost of large-scale production is much higher than the cost of small-scale farmers. Secondly, the egg has a high quality with bad price. Since there is no premium for good eggs, the price of egg may be much lower than the market price after producing large quantities of eggs, which leads to the slow development of the whole industrialization concentration. It should note that chicken seedlings are to increase the supply, and the epidemic is to reduce the production capacity. In 2013, the occurrence of H7N9 made the development of laying hens industry become not ideal in the next two years, therefore from 2013 to 2015, the GTCL in China has been negative growth, the situation has gradually begun to improve until 2016.

**Fig 2:**
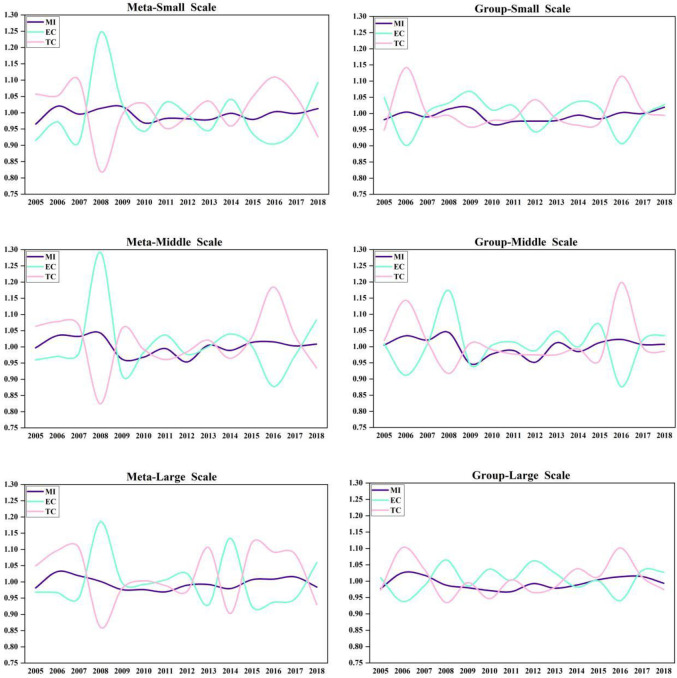
China’s various-sized GTCL and its decomposition target during 2004–2018.

As shown in Fig 2, at present, the overall level of GTCL in China is low. Obviously, laying hens’ feces treatment is a very important link in the process of laying hens breeding. However, in China, due to the imperfect laws and regulations related to the environmental protection, the lack of awareness of environmental governance, shortage of funds, immature feces treatment technology and other factors, the problem of feces processing is often ignored, resulting in more and more serious environmental pollution of laying hens’ feces [58].

Under the common frontier, EC increased suddenly in 2008. Under the group frontier, small-scale and large-scale EC values were normal, and middle-sized EC also increased abruptly. This is because at the end of 2007, China’s laying hens’ feed prices rose, which made the egg prices fall and farmers’ income decreased significantly. The popularization and application of technology level need time. Therefore, farmers began to improve the utilization of existing technology and management efficiency to expand their earnings. However, the distance from each region to feed is different, so on the condition of considering regional differences. the values of EC are different under the common frontier and group frontier. After the 21st century, there are three main modes of laying hens breeding in China: One is specialized chicken farm mode, the other is company + farmer mode, and the third is integrated production base mode. The larger the scale of farming, the higher the input rate of specialized equipment and technology. At present, the scale production system of laying hens in China is not perfect, which has not fully reached the stage of large-scale production. It remains in the transition stage from small-scale to large-scale. Therefore, the middle-scale GTCL is the highest in China.

**Fig 3:**
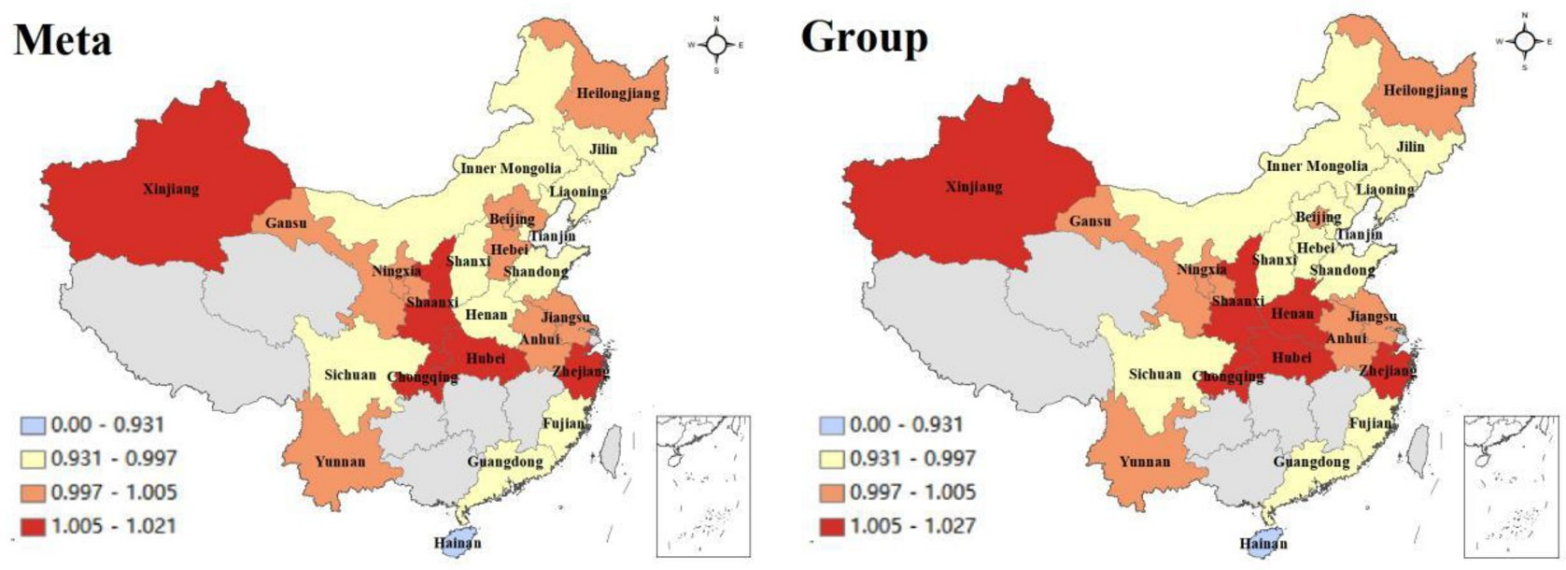
China’s GTCL in different provinces.

As can be seen from Fig 3, under the common frontier, the GTCLs of Hubei (0.99375), Inner Mongolia (0.99360), Guangdong (0.99107), Shanxi (0.98959), Gansu (0.97443) and Hainan (0.93197) were lower, while the GTCLs of Xinjiang (1.01047), Zhejiang (1.00958), Yunnan (1.00814), Anhui (1.00474), Jiangsu (1.00397) and Heilongjiang (1.00069) were higher and all above 1. Under the regional frontier, the GTCLs of Henan (0.98964), Hubei (0.98919), Jilin (0.98895), Shanxi (0.98455), Gansu (0.97503) and Hainan (0.93197) was lower, while the GTCLs of Jiangsu (1.01627), Xinjiang (1.01047), Zhejiang (1.00958), Yunnan (1.00814), Liaoning (1.00510) and Sichuan (1.00269) were higher and all above 1. No matter in the common frontier or group frontier, the GTCL of Hainan was ranked the last place. Gansu, Shanxi and Hubei are also at the bottom of the list, with negative growth. It demonstrates that these areas do not attach much attention to the problem of pollutant treatment in the process of laying hens breeding. From the perspective of laying hens breeding efficiency, the improvement of fecal treatment behavior will increase the breeding efficiency of laying hens, especially the improvement of the fecal cleaning mechanization level laying hens can effectively improve the breeding efficiency of laying hens and meet the requirements of the standardized development by improving the fecal treatment behavior. In addition, laying hens farming are highly dependent on feed input. In the early stage of industrial development, farmers are in the consideration of cost saving. In most cases, they use self-ingredients for feeding. With the marketization of feed supply, farmers’ feed purchase tends to be more market-oriented. The marketization of feed supply not only saves labor time, but also improves feed efficiency and ensures the quality of egg production.

**Fig 4:**
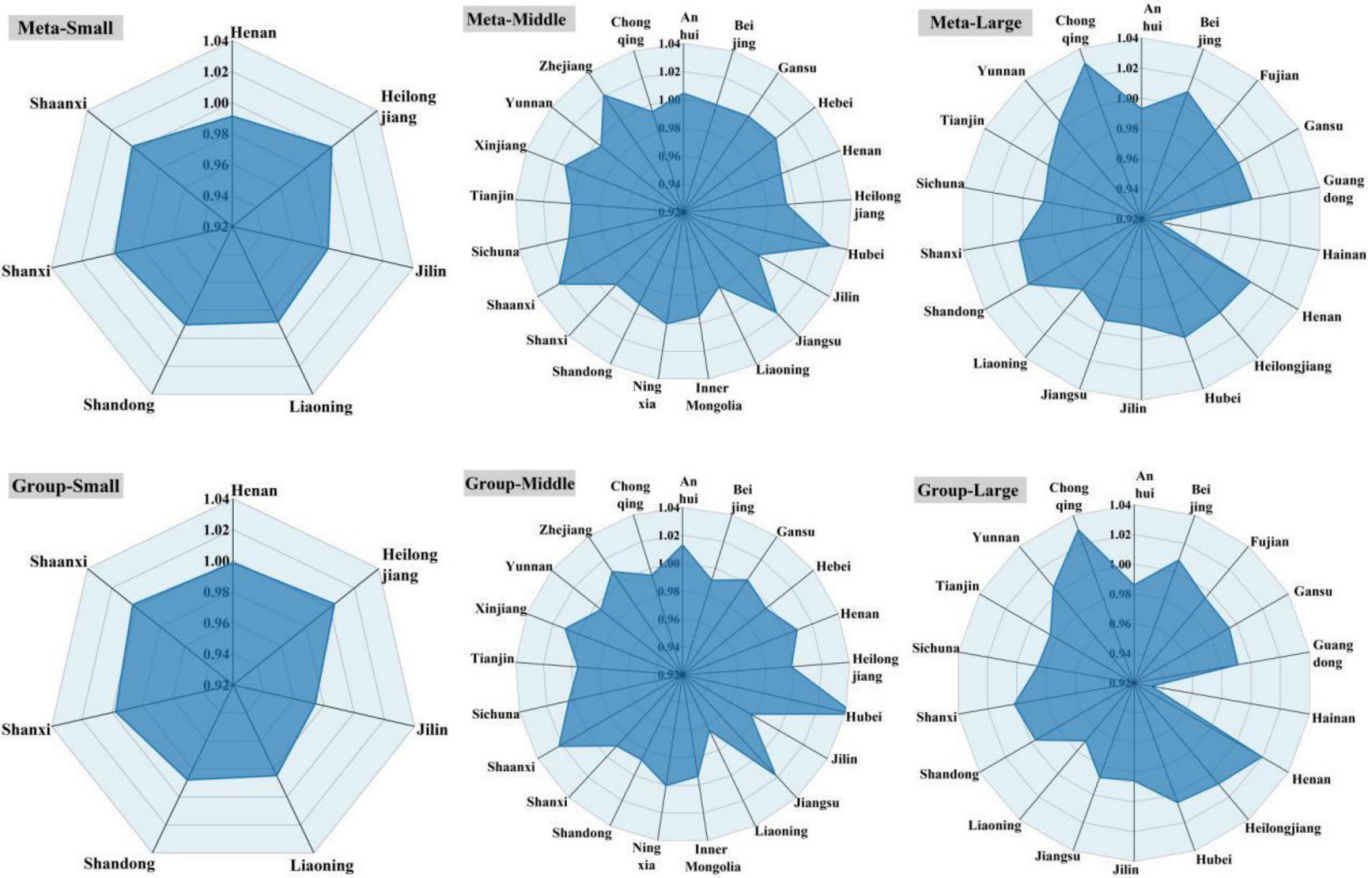
Three-scaled GTCL in different provinces.

As shown in Fig 4, under the common frontier, the GTCL of small-scale Shaanxi (1.00282) and Heilongjiang (1.00226) was greater than 1, the GTCL of middle-scale Hubei (1.02727), Shaanxi (1.02229), Zhejiang (1.02083) and Jiangsu (1.01742) was higher, and the GTCL of large-scale Chongqing (1.02973), Beijing (1.00962), Shandong (1.00656) and Yunnan (1.00431) was higher. Under the group frontier, the GTCL of small-scale Heilongjiang (1.00360) and Shaanxi (1.00282) was greater than 1, the GTCL of middle-scale Hubei (1.04656), Shaanxi (1.02229), Jiangsu (1.01808) and Anhui (1.01306) was higher, the GTCL of large-scale Chongqing (1.02973), Henan (1.01948), Heilongjiang (1.00848) and Beijing (1.00805) was higher. In general, the GTCL of small-scale, middle-scale and large-scale under the common frontier were 0.99378, 1.00132 and 0.99492 respectively. The GTCL of three scales under the group frontier were 0.99281, 1.00076 and 0.99368 respectively. With the continuous development of China’s laying hens farming industry, there are almost no provinces lacking eggs in China, including Gansu, which is now fully self-sufficient. At present, the provinces lacking eggs mainly include Qinghai, Tibet, Guangdong, Guangxi, Fujian and Hainan. The main reason why the south had not developed before was the shortage of corn raw materials [59]. All the raw materials are produced in the north. Now, all the imported raw materials are landing from the south to Hong Kong. There are also production management problems caused by high temperature and humidity, which can be completely solved by modern chicken houses. The solution of the raw materials problem and the development of modern chicken houses make Guangdong, Guangxi and other places develop rapidly in recent years.

**Fig 5:**
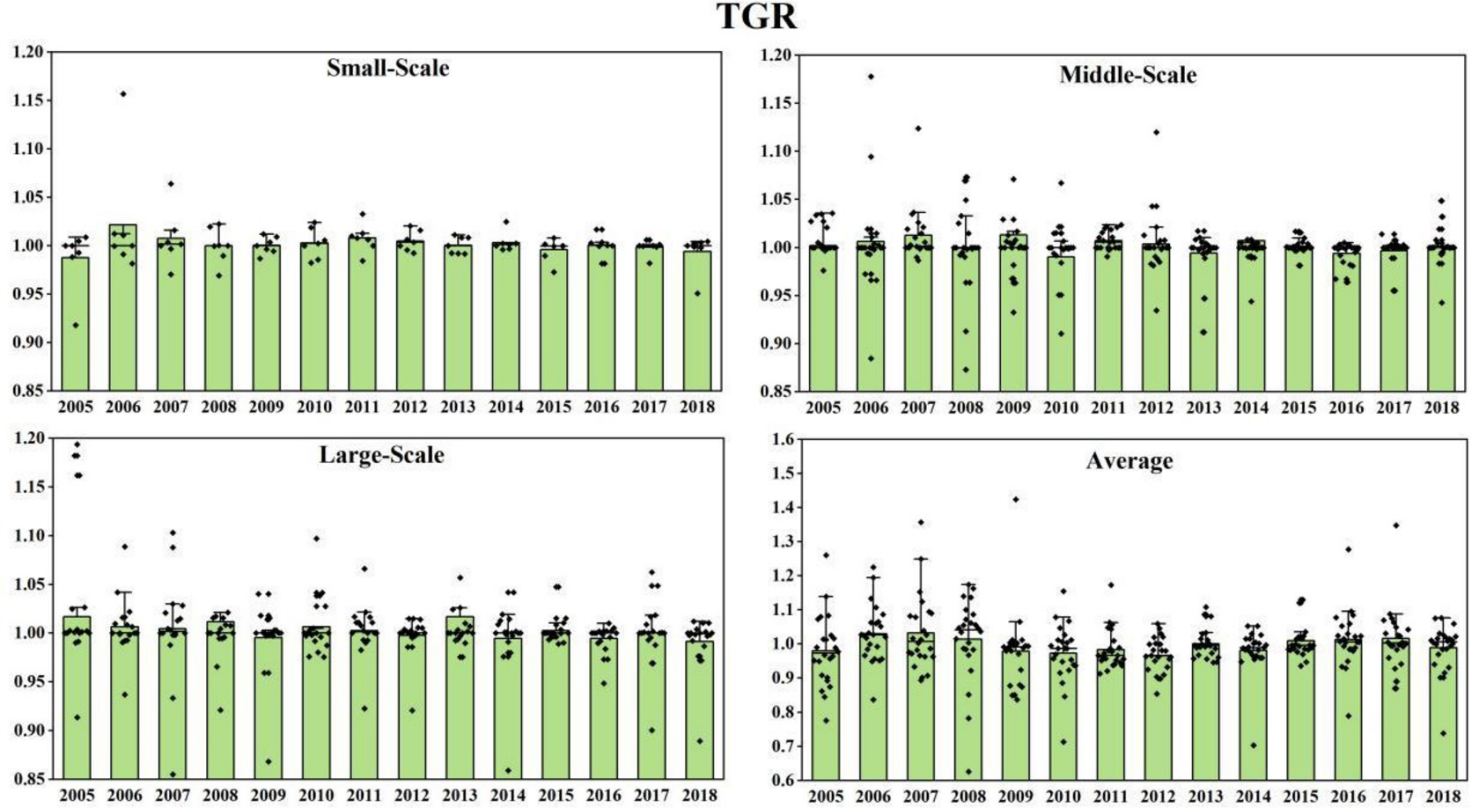
TGR in general and different scales during 2004–2018.

As shown in Fig 5, the TGRs of the three scales are relatively stable, with small- scale TGR (1.01582) was the farthest from 1 in 2006, middle-scale TGR (1.01602) was the farthest from 1 in 2009, large-scale TGR (1.01364) was the farthest from 1 in 2008, and overall TGR (1.00756) was the farthest from 1 in 2006. It indicated that small-scale laying hens breeding is still dominant in China. Due to the restrictions of capital and social environment, small-scale farmers are less likely to choose to relocate. How to achieve moderate scale farming under increasingly strict policy constraints is a major problem faced by farmers. At present, there are some phenomena in Chinese small-scale and middle-scale farming, such as low and unstable egg production rate, low egg quality and high incidence rate of chicken farms. Managers lack systematic operation ability and environmental awareness and investment. Due to the long-term decentralized breeding and lack of industry norms, there is a certain degree of overcapacity in the laying hens breeding industry, resulting in the waste of resources and the lower price trend of egg prices for a long time. At the same time, the rising labor costs, equipment costs and feed costs restrict the further development of the industry. China’s laying hens farming industry lacks certain international competitiveness. Therefore, it is the only way to realize the standardize of laying hens production in the future by further promoting our own excellent varieties, improving the mechanization of laying hens production, strengthening the monitoring of environmental parameters of chicken house, and improving the management ability of chicken farm.

### The change of GTCL in different regions

**Fig 6:**
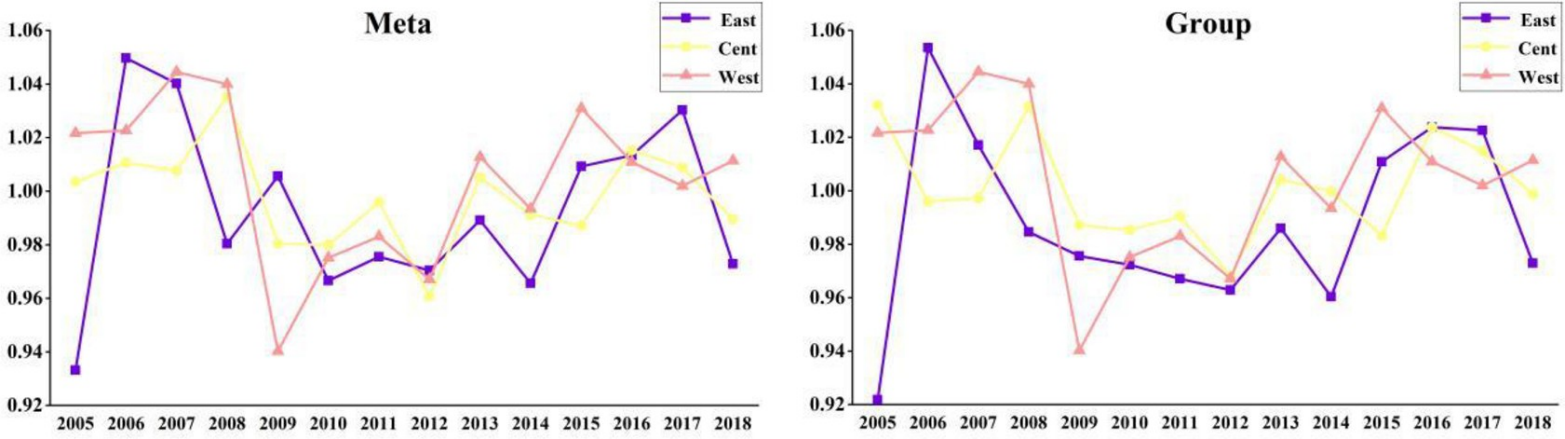
GTCL in three regions during 2004–2018.

It can be seen from Fig 6 that the changing trend of GTCL in each region is basically same under the common frontier and the group frontier. GTCL was low in 2005, which was influenced by the “2003 avian influenza” epidemic in China. Since 2008, the laying hens’ industry has entered a stage of self-integration, and the impact of the “avian influenza” incident has accelerated this process. At present, the biggest bottleneck of the healthy development of China’s laying hens breeding industry is the frequent occurrence of diseases. On the one hand, the diseases will cause significant economic losses to farmers, leading to a sharp rise in breeding costs. On the other hand, it will lead to a decline in the quality of eggs, resulting in hidden dangers in food quality and safety, and an impact on consumer psychology. The main reasons for the frequent epidemic of laying hens are the lack of professional skills, the lack of corresponding testing equipment, the single breed of laying hens, the primary abuse, the lack of awareness of comprehensive disease prevention and control, and the illegal feed additives. Obviously, these factors lead to the sluggish sales of laying hens products, and have an extremely negative impact on the development of laying hens’ industry. Since 2014, a large number of social capitals has entered the laying hens breeding industry, the number of large-scale chicken farms has increased, and the process of industrial scale development has been promoted rapidly. However, the resulting biological risks and environmental risks are still the key and difficult points in the process of large-scale breeding. Under the common frontier, the average GTCLs of the eastern region, central region and western region were 0.99196, 0.99611 and 0.99795 respectively. Under the regional frontier, the average GTCLs of three regions were 0.99374, 0.99136 and 0.99832 respectively. In China, the eastern region is densely populated with small land area, and lacks natural conditions for laying hens breeding; the central region is located in the middle of China, with superior geographical location and low transportation cost; the western region has low labor cost [60], large area, open terrain and superior natural conditions. Therefore, the GTCL of China in western region is higher than the GTCL in central region and eastern region in most years.

**Fig 7:**
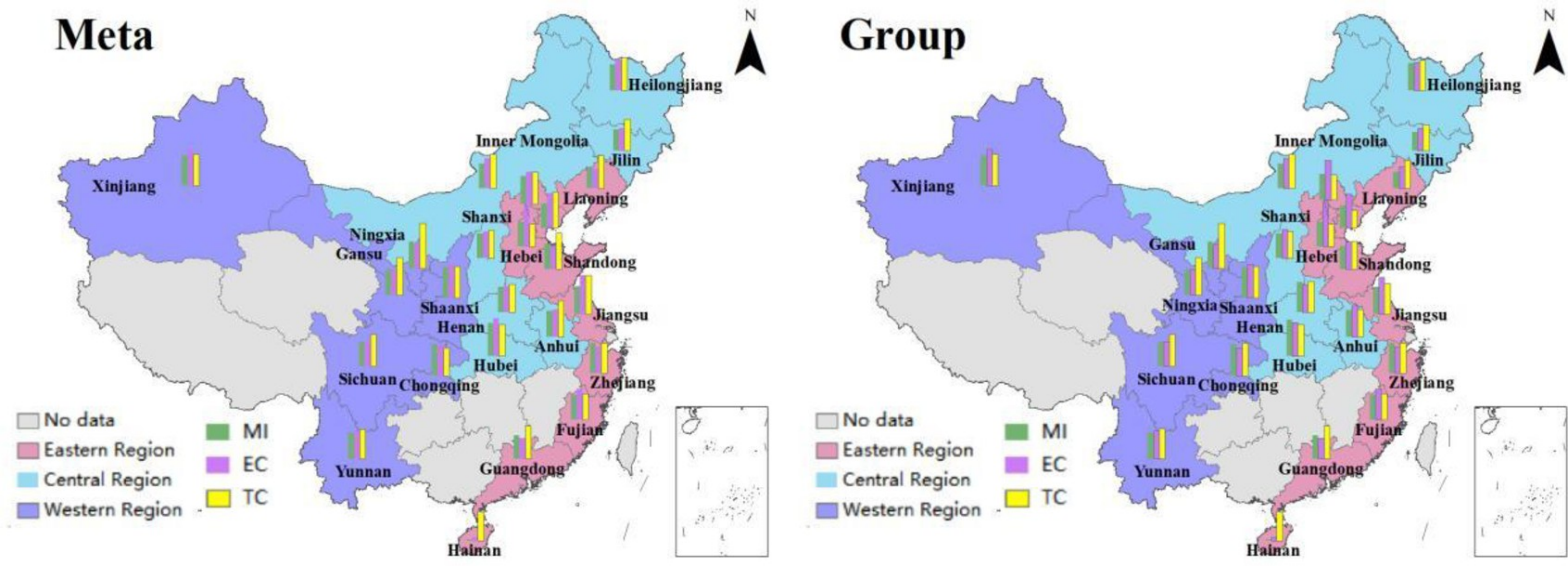
Average GTCL and its decomposition indexes in three regions.

As shown in Fig 7, under the common frontier, in the eastern region, the GTCL of Zhejiang (1.00958), Jiangsu (1.00397) and Tianjin (1.00066) were higher, while the GTCL of Guangdong (0.99107) and Hainan (0.93197) were lower, which was lower than the average value of eastern region (0.99196). In the central region, the GTCL of Anhui (1.00474) and Heilongjiang (1.00069) were higher, which was higher than the average value of central region (0.99611), while the GTCL of Hubei (0.99375), Inner Mongolia (0.99360) and Shanxi (0.98959) were lower. in the western region, the GTCL of Xinjiang (1.01047), Yunnan (1.00814) and Ningxia (1.00024) were higher, and the GTCL of Shaanxi (0.99401) and Gansu (0.97443) were lower, which was lower than the average value of western region (0.99795). At present, the capital of farmers’ breeding is relatively free entry and exit. When the price of eggs goes up and the expected income is obvious, the farmers will buy chicken seedlings for breeding. When the price of eggs drops, the farmers expect laying hens breeding will be loss and eliminate the laying hens in time. The free entry and exit of laying hens breeding is not conducive to the stable supply and demand in egg market, but objectively promotes the large-scale breeding process. The development of large-scale breeding must follow the principle of environmental priority and economic balance. On the basis of ensuring the environment, the economic benefits of laying hens breeding should be improved, so as to protect the enthusiasm of farmers. The government should actively play the role of overall planning and comprehensive coordination, and promote the appropriate scale of laying hens’ industry and the comprehensive development of related industries. At the same time, the government should also strive to control and reduce the pollution, and protect the living and production environment.

**Fig 8:**
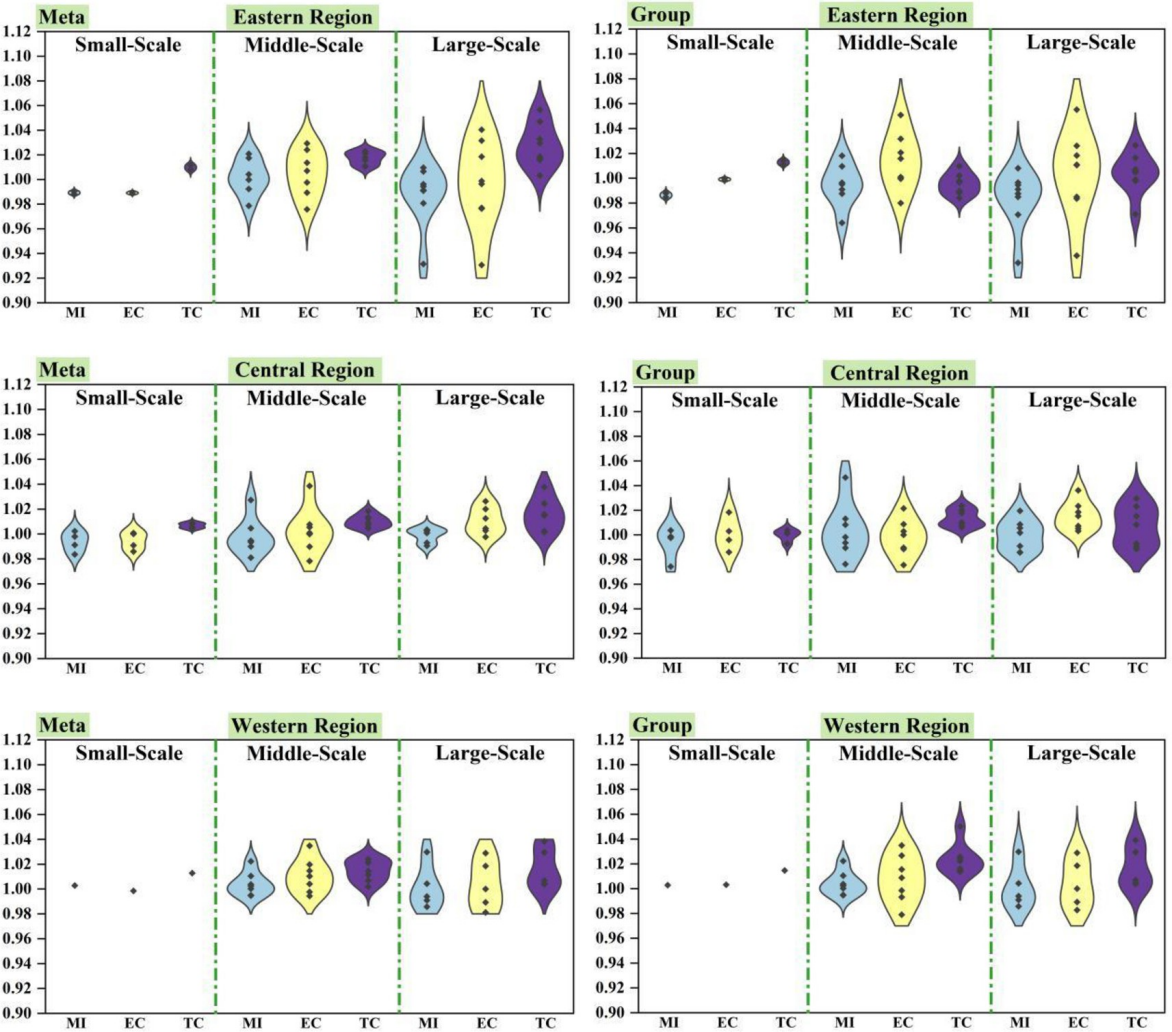
Different sized average GTCL and its decomposition targets in three regions.

As shown in Fig 8, under the common frontier, the GTCL of small-scale, middle-scale and large-scale in the eastern region were 0.98928, 1.00188 and 0.98766 respectively. In the central region, the GTCL were 0.99376, 0.99797 and 0.99893, respectively. In the western region, the GTCL were 1.00282, 1.00410 and 1.00344 respectively. Obviously, the GTCL of three scales in the western region were all greater than 1, the GTCL of middle-scale in the eastern region was greater than 1, and the GTCL of three scales in the central region were all less than 1. Under the regional frontier, the GTCL of three scales in the eastern region were 0.98632, 0.99456 and 0.98319 respectively. In the central region, the GTCL were 0.99356, 1.00362 and 1.00116 respectively. In the western region, the GTCL were 1.00282, 1.00410 and 1.00344 respectively. Similarly, the GTCL of three scales in western region were greater than 1, but the GTCL of middle-scale and large-scale in central region were greater than 1, and the GTCL of three scales in eastern region were less than 1. It showed that the development of the western region was better, it should pay more attention to ecological effects [61], and the natural breeding conditions in the western China were superior. At the same time, in most cases, middle-scale GTCL is higher than small-scale and middle-scale. The cost-benefit ratio of small-scale breeding is generally higher than that of middle-scale and large-scale breeding. However, the profit margins of three kinds of scale laying hens are not high, which indicates that the profit earned by laying hens is far less than the total cost of investment. In particular, the cost input in the early stage of large-scale breeding is larger and the profit is lower.

**Fig 9:**
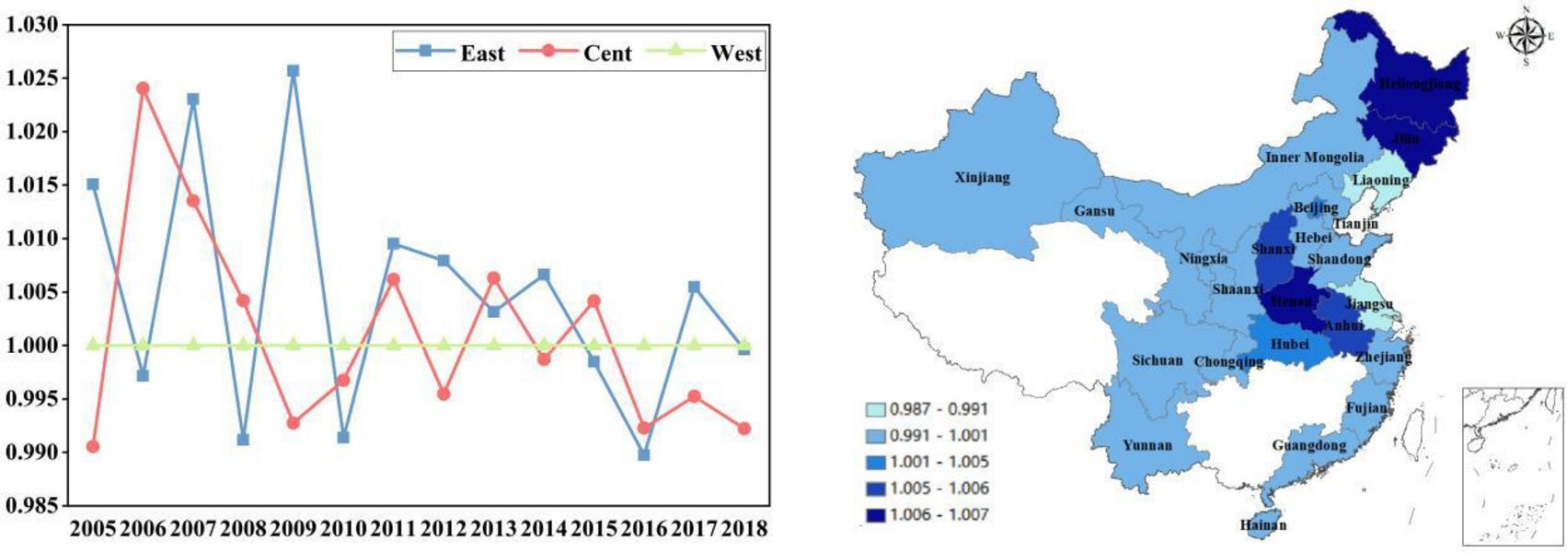
Average TGR in different regions.

As shown in Fig 9, the TGR of the western region was 1 from 2004 to 2018, indicating that the development of laying hens breeding industry in the western region was better, and the government attached great importance to the treatment of feces. The TGR in the eastern region and central region fluctuated greatly, with the average TGR of the eastern region is 1.00457, and the average TGR of the central region is 1.00087. It showed that the technological level in the eastern region and the central region was advanced, which was consistent with the actual situation in China. The national average TGR was 1.00216. Although some large-scale breeders have realized the importance of laying hens feces cleaning, the level of laying hens feces cleaning of large-scale breeders in China is still low, especially the mechanization level of cleaning is low. Compared with other investment, the investment of laying hens breeding is often lack of technical, financial and policy support, which makes the equipment investment level and the mechanization level of cleaning low. It is not conducive to the healthy breeding of laying hens. The education level of farmers, the number of organic fertilizer plants, the distance from the township government, and the original way of laying hens feces utilization are all important factors affecting the way of laying hens feces use. Therefore, it should improve the utilization of laying hens feces by improving the quality of householders and the breeding environment. Through the high level of laying hens feces treatment, it can more effectively protect egg production, thus improving breeding income.

## Conclusions and policy suggestions

Based on the SBM model, this paper constructs the MML index by considering the unexpected output, and it calculates China’s GTCL from 2004 to 2018 and draws the following conclusions: (1) Regardless of the common frontier or group frontier, the GTCL shows a large spatial and temporal differentiation characteristic. Compared with the eastern region and central region, the western region has advantages in GTCL. (2) The GTCL generally shows a downward trend, but an upward trend in recent years. The GTCL declined by 0.333% under the common frontier and 0.425% under the group frontier on average. (3) Middle-scale GTCL has advantages compared with the small-scale and the large-scale. Whether common frontier or group frontier, middle-scale GTCL was higher than large-scale and small-scale, and large-scale GTCL was higher than small-scale.

Combined with the reality of China’s laying hens breeding industry, the following policy implications are put forward:

1. Improve the mechanization level of laying hens breeding and promote the appropriate scale of laying hens’ industry by optimizing the rational allocation with elements. The continuous improvement of China’s mechanization level is an important condition for large-scale development. First of all, it is necessary to promote scientific innovation of breeding equipment, encourage the upgrading and transformation of specialized equipment, such as fully automatic and highly applicable feeding equipment, environmental control equipment, epidemic prevention and control equipment, and other specialized equipment, so as to promote the large-scale development of the industry. Secondly, it needs to promote the socialization service level of key links of mechanized breeding, solve the problem of machinery purchase cost of small-scale and middle-scale farmers, and encourage the sharing and public use of breeding equipment in conditional areas. Finally, it is necessary to improve the subsidy standard and strength for large-scale farmers to purchase fully automatic machinery, and comprehensively promote the development of laying hens breeding mechanization. Optimize the rational allocation of machinery and labor input to achieve economies of scale and promote the organic connection between small farmers and modern agriculture.
2. The government and farms should pay more attention to the treatment of wastes, such as feces. In China, GTCL overall shows a downward trend. Therefore, it is necessary to attach great importance to the impact of waste treatment on laying hens breeding. Government should raise awareness of the impact on pollution by means of publicity and education. Simultaneously, government should increase subsidies for livestock and poultry waste treatment machinery, play laying hens breeding reasonably, and establish a demonstration base for laying hens waste treatment. In addition, it is crucial to strictly plan the range of forbidden zone, restricted zone and suitable zone, and strictly control the scale of livestock and poultry breeding in the breeding area. The regional farms should be reasonably guide to upgrade the sewage facilities.
3. Improve the level of prevention and control of avian influenza. In order to improve the production efficiency of laying hens, it is necessary to control the frequent occurrence of epidemic diseases of laying hens and reduce the impact of egg quality and safety hazards on consumers. Therefore, in the process of laying hens breeding, it needs to strengthen the professional skills and management level of laying hens farmers, try to be consistent with the breeding varieties, and strengthen the awareness of comprehensive prevention and control of epidemic diseases, so as to promote the development of laying hens breeding industry, reduce the epidemic diseases loss of farmers, and narrow the government’s investment in epidemic compensation. In addition, it should note that the establishment of improved breeding system can improve the production performance of laying hens persistently and efficiently. It needs to establish a prevention and control fund of avian influenza, vigorously develop policy-oriented poultry insurance, support existing commercial insurance companies to carry out policy poultry insurance services through tax incentives and other measures, so that poultry farmers can be insured.
